# Paraneoplastic Neuromyelitis Optica Spectrum Disorder Associated With Lung Adenocarcinoma: A Case Report

**DOI:** 10.3389/fmed.2021.743798

**Published:** 2022-01-17

**Authors:** Carlo Maiorca, Federica Moret, Valentina Martines, Daniele Tramontano, Maria Alessia Papassifachis, Simone Bini, Claudia Caramazza, Mario Fontana, Piernatale Lucia, Maurizio Inghilleri

**Affiliations:** ^1^Department of Translational and Precision Medicine, Policlinico Umberto I, Sapienza University of Rome, Rome, Italy; ^2^Department of Human Neurosciences, Sapienza University of Rome, Rome, Italy

**Keywords:** neuro-oncology, NMOSD, demyelination, adenocarcinoma, aquaporin-4

## Abstract

Neuromyelitis Optica spectrum disorder is an inflammatory demyelinating disease affecting the central nervous system (CNS), characterized by triad optic neuritis, transverse myelitis, and area postrema syndrome. Antibodies directed against aquaporin-4 (AQP-4), a water channel expressed on the astrocytic membrane, are supposed to play a pathogenic role and are detected in ~80% of cases. Clinical signs of Neuromyelitis Optica spectrum disorder (NMOSD) in elderly patients should arouse the suspicion of paraneoplastic etiology. In this article, we discussed a case of a 76-year-old woman with a 2-month history of confusion, dysarthria, and progressive bilateral leg weakness. A whole-body CT scan showed a neoformation of 5 cm in diameter in the median lobe infiltrating the mediastinal pleura. The tumor had already spread to both the upper and lower right lobes, parietal pleura, and multiple lymph nodes. Pleural cytology revealed adenocarcinoma cells. The brain MRI documented hyperintense alteration in fluid-attenuated inversion recovery (FLAIR) images, involving the anterior portion of the corpus callosum and the periependymal white matter surrounding the lateral ventricles, with mild contrast enhancement on the same areas and meningeal tissue. T2-weighted spinal cord MRI sequences showed extended signal hyperintensity from bulbo-cervical junction to D7 metamer, mainly interesting the central component and the gray matter. Cerebrospinal fluid analysis revealed no neoplastic cells. Serum AQP-4 immunoglobulin (IgG) antibodies were found. Meanwhile, the patient rapidly developed progressive paraparesis and decreased level of consciousness. High-dose intravenous methylprednisolone therapy was started but her conditions rapidly deteriorated. No other treatment was possible.

## Introduction

Neuromyelitis Optica spectrum disorder is an inflammatory demyelinating disease affecting the central nervous system (CNS), characterized by the triad optic neuritis, transverse myelitis, and area postrema syndrome, eventually associated with other neurologic manifestations (1–3). Antibodies directed against aquaporin-4 (AQP-4), a water channel expressed on the astrocytic membrane, are supposed to play a pathogenic role (4, 5) and are detected in ~80% of cases (6). Neuromyelitis Optica spectrum disorder (NMOSD) can manifest in the context of systemic autoimmune diseases, such as Systemic Lupus Erythematosus and Sjogren syndrome (6). As part of immune dysregulation, some cases of NMOSD are reported as a paraneoplastic manifestation (7–11). Herein, we discuss a case of a 76-year-old woman with NMOSD associated with metastatic lung adenocarcinoma.

## Case Description

**Figure 1:**
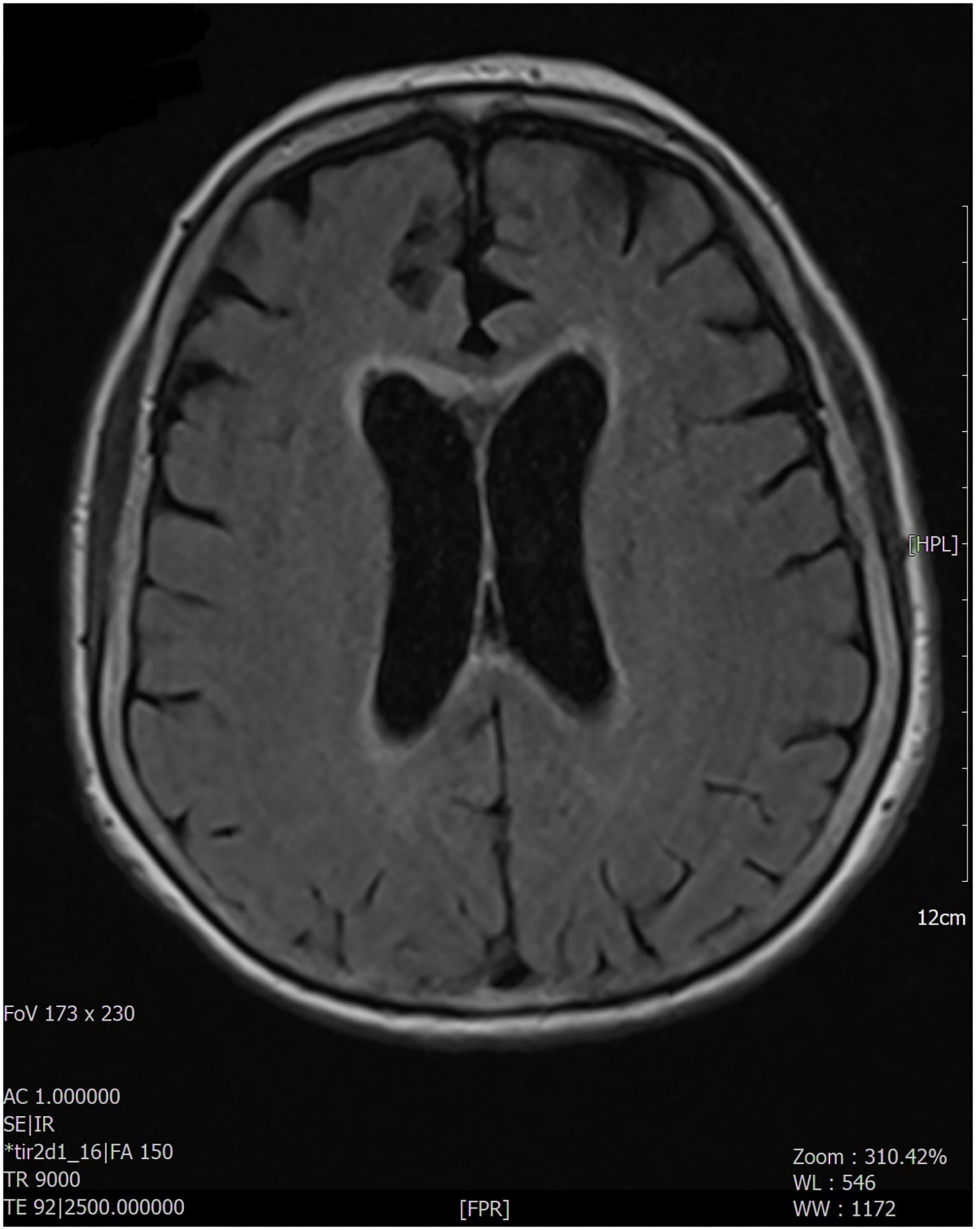
The MRI examination documented the presence, at the level of the brain, of hyperintense alteration, in FLAIR images, involving the anterior portion of the corpus callosum and the peri-ependymal white matter at the level of the lateral ventricles. The remaining brain areas of greater expression of aquaporin 4 (diencephalon, midbrain, a pons) did not appear to be affected.

**Figure 2:**
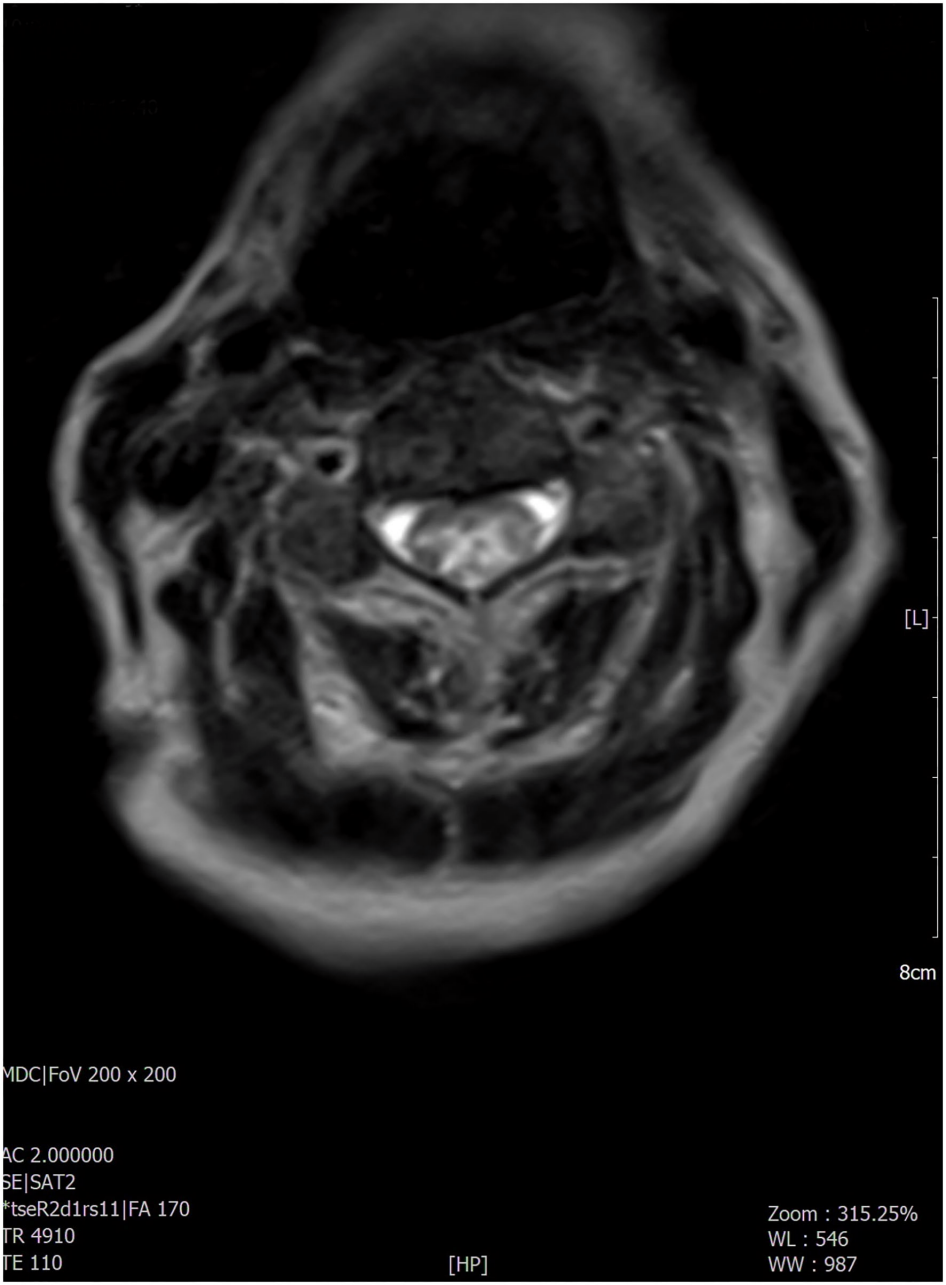
The most significant findings were found at the level of the cervico-dorsal cord with evidence, in the T2-weighted sequences, of extended signal hyperintensity which mainly concerned the central component and in particular the gray matter.

**Figure 3:**
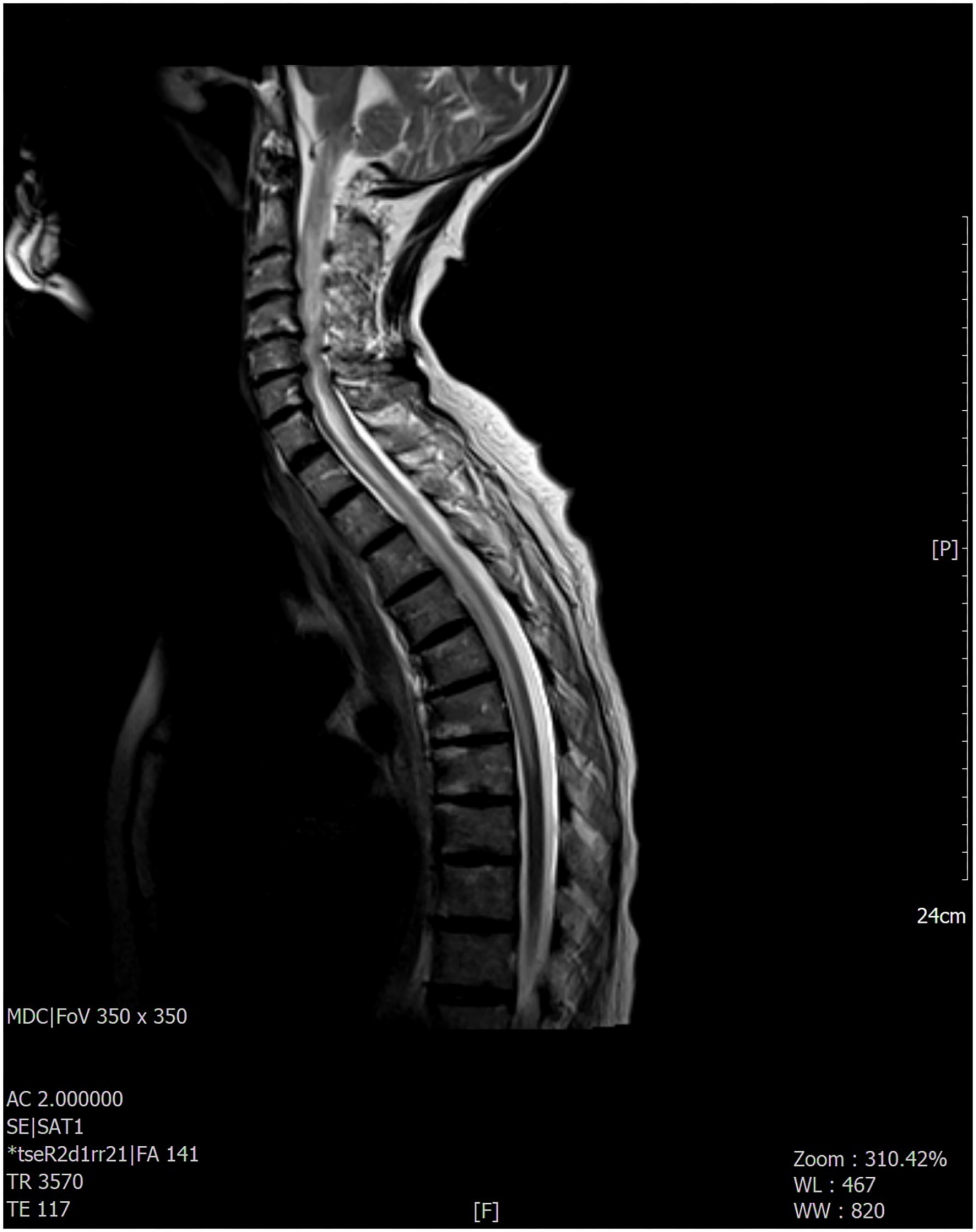
The most significant findings were found at the level of the cervico-dorsal cord with evidence, in the T2-weighted sequences, of extended signal hyperintensity which mainly concerned the central component as best evident in the axial acquisition.

A 76-year-old woman with no specific medical, smoking, or family history accessed our emergency department because of a 2-month history of confusion, dysarthria, and progressive bilateral leg weakness. She also reported a recent onset of blurred vision, diplopia, weakness of the left upper limb, and two episodes of sudden fall. On neurologic examination, the patient was confused and partially space-time oriented; strength and sensitivity examinations were difficult to test for the patient's partial collaboration, however nociceptive stimulation caused flexion response at four limbs. Deep tendon reflexes were absent in the four limbs, while Babinski's sign was present bilaterally. A decreased vesicular murmur of the right hemithorax was found. Routine blood tests only showed high levels of C-reactive protein (6.9 mg/dl, normal values < 0.5). The chest X-ray revealed unilateral massive right side pleural effusion and a right basal paracardiac opacity. A whole-body CT scan showed a neoformation of 5 cm in diameter in the median lobe infiltrating the mediastinal pleura. The tumor had already spread to both the upper and lower right lobes, parietal pleura, and multiple lymph nodes (right hilum, right internal mammary, right paratracheal and, subcarinal chains); no other distant metastasis was found. Thoracentesis was performed, in which the pleural cytology revealed adenocarcinoma cells BRAF/Epidermal Growth Factor Receptor (EGFR)/KRAS/Activin-Like Kinase (ALK)/ROS-1 wildtype, thyroid transcription factor-1 (TTF-1)/napsin-A positive, p40/calretinin negative, positive expression of programmed death-ligand 1 (PDL-1) with tumor proportion score (TPS) score 80%. A non-contrast brain CT scan showed mild cortical-subcortical atrophy. The brain MRI documented hyperintense alteration in fluid-attenuated inversion recovery (FLAIR) images, involving the anterior portion of the corpus callosum and the periependimal white matter at the level of the lateral ventricles (Figure 1) with mild contrast enhancement on the same areas and on meningeal tissue widely. The most significant findings were found at the level of the spinal cord with evidence, in the T2-weighted sequences, of extended signal hyperintensity from bulbo-cervical junction to D7 metamer, which mainly concerned the central component and in particular the gray matter (Figure 2), as best evident in the axial acquisition (Figure 3), associated with cord swelling and contrast enhancement on the same level. To exclude a neurologic disorder related to a neoplastic condition such as CNS lymphoma, meningeal carcinomatosis, or paraneoplastic condition, a lumbar puncture was performed; cerebrospinal fluid analysis revealed <10 cells/mcL, increased protein level (366 mg/dl; normal range 15–45), normal glucose level (71 mg/dl; normal range 50–80) and no neoplastic cells. No serum anti-neuronal antibodies [anti-Amphisine, anti-CV2.1, paraneoplastic antigen Ma2 (anti-PNMA2), anti-Ri, anti-Yo, and anti-Hu] were detected, while serum aquaporin-4 (AQP-4) immunoglobulin (IgG) antibodies were found. Meanwhile, the patient's neurologic conditions worsened; she developed progressive paraparesis and decreased level of consciousness. The spinal and cerebral MRI findings, associated with the presence of AQP-4 IgG antibodies, are suggestive of paraneoplastic NMOSD. High-dose intravenous methylprednisolone therapy was started but the patient's condition further and rapidly deteriorated, so that no other treatment was possible. The patient died a few days later.

## Discussion

Neuromyelitis Optica spectrum disorder (NMOSD) is a group of demyelinating, autoimmune disorders affecting the central nervous system. The worldwide prevalence of NMOSD ranges from 0.5 to 10 cases per 100,000 (12). NMOSD is usually diagnosed in young adults (35–45 years of age) with a female predominance (13). In most cases, NMOSD is associated with autoantibodies targeting AQP-4, a transmembrane protein present on the surface of astrocyte projections where it regulates water movement between the cerebrospinal fluid, blood, and brain. In ~10–20% of patients with clinical signs of NMOSD serum anti-AQP-4 are absent and other autoantibodies [CV2/collapsin response mediator protein 5 (CRMP5), anti-neuronal nuclear antibody type-1 (ANNA-1), and amphiphysin] might be responsible for central nervous system damage (14). The antibody production is probably triggered by dysregulation of the immune response against neoplastic cells and some case reports have demonstrated AQP-4 expression in tumor tissue (15), but the exact underlying mechanisms are still unknown. Symptoms suggestive of NMOSD are optic neuritis, sometimes bilateral and involving optic chiasm with severe visual loss or visual field defect; longitudinally extensive transverse myelitis (LETM) involving predominantly the gray matter with cord swelling, with clinically acute myelitis and complete spinal cord syndrome; area postrema syndrome, with intractable hiccups, nausea, and vomiting (2). There could be other neurological manifestations, including narcolepsy and other diencephalic symptoms, brainstem syndromes, and cognitive dysfunction (6).

Clinical signs of NMOSD in elderly patients, especially of subacute onset, should arouse the suspicion of paraneoplastic etiology (16). Indeed, several cancers are known to be associated with NMOSD. In 2021, Shahmohammadi et al. performed a comprehensive literature search for all types of cancers associated with NMOSD (16). The authors described 62 cases that met the Paraneoplastic Neurologic Syndromes (PNS) Euro network criteria (17). NMOSD preceded cancer diagnosis only in 19/62 cases (30.6%). The most-reported cancers in women (*n* = 49, 79.03%) were breast (*n* =11, 22.5%) and genitourinary (*n* = 11, 22.5%), respectively. Overall, the authors reported nine (14%) lung adenocarcinoma (six women and three men, median age 61). Interestingly, seven out of nine patients with lung adenocarcinoma presented with NMOSD as the first manifestation of cancer. Other relevant information can be found in Table 1.

**Table 1:** Shahmohammadi et al. (16) cancer population (*n* = 62).

**Most reported organs in cancer population (*n* = 62)**	**Number of cases**
-Genitourinary	14 (22.3%)
-Breast	12 (19.4%)
-Lung	12 (19.4%)
(adenocarcinoma)	9 (14.5%)
-Gastrointestinal	7 (11.3%)
- Hematology	6 (9.7%)
**Most reported cancer in women (*n*= 49)**	
-Breast	11 (22.5%)
- Genitourinary	11 (22.5%)
**Most reported cancer in men (*n* = 13)**	
-Lung	3 (23.1%)
-Genitourinary	3 (23.1%)
- Hematology	3 (23.1%)
NMOSD preceding cancer diagnosis	19 (30.6%)
NMOSD preceding lung adenocarcinoma diagnosis	7/9 (78%)
Mean age in cancer population	53.83 ± 15.58 years old
Median in age in lung adenocarcinoma population	61 (37–72) years old

In general, the prognosis of paraneoplastic NMOSD significantly depends on the type and stage of tumors. Treatment options include oncologic (surgery, chemo- and radiotherapy) and immunosuppressive therapies (high-dose intravenous steroids, intravenous immunoglobulins, azathioprine, rituximab, etc.) (18).

Here, we reported a case of a 76-year-old woman with paraneoplastic NMOSD associated with IV-stage lung adenocarcinoma. The patient firstly manifested drowsiness and motor speech disorder, later developing progressive left upper limb and bilateral leg weakness, blurred vision, diplopia, and reduced level of consciousness. Neuroimaging showed an LETM from cervico-bulbar junction to dorsal spinal cord, predominantly involving the central gray matter and a periventricular hyperintensity on FLAIR sequences with widespread meningeal contrast enhancement.

This radiological aspect is suggestive of NMOSD, particularly periependimal and longitudinal extensive central spinal cord lesions with cord swelling and extension to the medulla, due to typical distribution of AQP-4 on the surface of astrocyte projections in these areas (2, 3, 19), while the remaining brain areas of greater expression of AQP-4 (diencephalon, midbrain, and pons) did not appear to be affected.

This clinical and radiologic picture, along with CSF results, anti-AQP-4 IgG positivity in the absence of other paraneoplastic markers, is consistent with NMOSD diagnosis, according to the most recent consensus criteria (2).

Surgical treatment was not an option because of the advanced oncologic disease. The patient's conditions rapidly deteriorated; no improvement was detected with high-dose intravenous methylprednisolone and no other therapies were possible to start.

## Conclusion

Our findings increase the recognition that AQP4-antibody-positive NMOSD, especially in elderly patients, should suggest the presence of underlying cancer. Lung adenocarcinoma is not one of the most frequently occurring cancers in paraneoplastic NMOSD cases and very often the neoplasm is already advanced at the moment of diagnosis (16). In lung adenocarcinoma, despite many other tumors, NMOSD symptoms usually precede cancer diagnosis (16). We believe our case is particularly interesting because of the extremely rapid clinical deterioration thus underlying the importance of a rapid diagnosis. Unfortunately, in lung adenocarcinoma presenting with NMOSD, the advanced oncologic disease usually precludes any resolutive treatment, and support therapy is essential for early counteracting the neurological worsening.

## Data Availability Statement

The original contributions presented in the study are included in the article/supplementary material, further inquiries can be directed to the corresponding author.

## Ethics Statement

Written informed consent was obtained from the individual(s) for the publication of any potentially identifiable images or data included in this article.

## Author Contributions

CM wrote the first draft of the manuscript. FM, PL, and MI wrote sections of the manuscript. MF, PL, and MI contributed to manuscript revision. All authors contributed to the conception and design of the study and read and approved the submitted version.

## Conflict of Interest

The authors declare that the research was conducted in the absence of any commercial or financial relationships that could be construed as a potential conflict of interest. The handling editor declared a shared affiliation with the authors at the time of the review.

## Publisher's Note

All claims expressed in this article are solely those of the authors and do not necessarily represent those of their affiliated organizations, or those of the publisher, the editors and the reviewers. Any product that may be evaluated in this article, or claim that may be made by its manufacturer, is not guaranteed or endorsed by the publisher.
